# Mapping canopy traits over Québec using airborne and spaceborne imaging spectroscopy

**DOI:** 10.1038/s41598-023-44384-0

**Published:** 2023-10-11

**Authors:** Thomas Miraglio, Nicholas C. Coops, Christine I. B. Wallis, Anna L. Crofts, Margaret Kalacska, Mark Vellend, Shawn P. Serbin, Juan Pablo Arroyo-Mora, Etienne Laliberté

**Affiliations:** 1 Integrated Remote Sensing Studio, Department of Forest Resources Management, University of British Columbia, 2424 Main Mall, Vancouver, BC V6T 1Z4 Canada; 2 Département de Biologie, Université de Sherbrooke, Sherbrooke, QC Canada; 3 Applied Remote Sensing Lab, Department of Geography, McGill University, Montréal, QC H3A 0G4 Canada; 4 Environmental and Climate Sciences Department, Brookhaven National Laboratory, Upton, NY 11973 USA; 5 Flight Research Laboratory, National Research Council of Canada, Ottawa, ON K1A 0R6 Canada; 6 Département de Sciences Biologiques, Institut de Recherche en Biologie Végétale, Université de Montréal, Montréal, QC H3A 0G4 Canada

**Keywords:** Forest ecology, Biodiversity, Forestry

## Abstract

The advent of new spaceborne imaging spectrometers offers new opportunities for ecologists to map vegetation traits at global scales. However, to date most imaging spectroscopy studies exploiting satellite spectrometers have been constrained to the landscape scale. In this paper we present a new method to map vegetation traits at the landscape scale and upscale trait maps to the continental level, using historical spaceborne imaging spectroscopy (Hyperion) to derive estimates of leaf mass per area, nitrogen, and carbon concentrations of forests in Québec, Canada. We compare estimates for each species with reference field values and obtain good agreement both at the landscape and continental scales, with patterns consistent with the leaf economic spectrum. By exploiting the Hyperion satellite archive to map these traits and successfully upscale the estimates to the continental scale, we demonstrate the great potential of recent and upcoming spaceborne spectrometers to benefit plant biodiversity monitoring and conservation efforts.

## Introduction

Characterizing the functional diversity of vegetation is crucial to advance biodiversity conservation efforts^1^. Vegetation traits, such as the biochemical properties of leaves, are of prime importance for effective monitoring of ecosystem functioning and diversity^2,3^, the global carbon cycle^4,5^, and forecasting climate change. Indeed, terrestrial biosphere models categorize similar vegetation into groups, or plant functional types (PFT), to describe common physical, biological, and physiological properties of plants to simplify the representation of the large degree of global plant diversity^6^. Because of this, PFT parametrization has a significant influence on the modelled cycling of carbon, water, and energy in terrestrial ecosystems^7^. Likewise, improving the modelling of plant nitrogen and phosphorus cycles by incorporating trait information is critical to better predict the net primary productivity of vegetation^8,9^ and allow for better projections of global climate change.

Several vegetation traits have also been established as essential biodiversity variables (EBV)^10^ and may be accessible using remote sensing through various data products. While proximal remote sensing enables the retrieval of leaf traits, such as the leaf chlorophyll content or the specific leaf area, at the individual level, airborne and satellite-based remote sensing allows the estimation of canopy traits including leaf traits upscaled at the community level, the leaf area index (LAI), or vegetation cover, over large areas^11^. More specifically, satellite-based remote sensing of canopy traits is expected to play a key role in ongoing global vegetation monitoring efforts^12^. However, while remote sensing has been largely used to estimate canopy traits^13–16^, most studies have been undertaken at the landscape scale, using either airborne imaging spectrometers or multispectral satellite sensors. These studies often use models calibrated using field data acquired concomitantly with the remote sensing acquisitions^16–18^ or sensors for which trait retrieval methods had already been pre-calibrated for the specific ecosystem^19^. Upscaling local estimates of vegetation traits to the landscape, continental and global levels remains a challenge^20^, and the full potential of remote sensing to further global ecological studies has yet to be realized.

Direct global estimations of some canopy traits have been shown to be possible. Continental and global-scale maps have been obtained by combining data from multispectral satellite sensors with trait databases built from field measurements^21,22^, or with radiative transfer models (RTM)^23^. However, due to the inability of multispectral sensors to resolve narrow spectral features, and the sparsity of trait databases, some canopy traits may prove to be very poorly estimated or simply inaccessible. In 2000, NASA launched the EO-1 platform, which contained the Hyperion instrument, becoming the first publicly available imaging spectrometer in space. Since then, only a limited number of instruments have been launched, including HISUI on the ISS, PRISMA, and EnMAP. Imaging spectrometers are becoming more common as spaceborne remote sensing observation platforms^24^, with a number of private satellites and national space agencies missions planned.

Imaging spectrometers, because of their narrow bandwidths and large number of contiguous spectral bands, provide rich spectral information and can be applied to a greater variety of ecological questions^25^. Hyperion acquired images from 2000 to 2017 and its datasets are freely available, and despite the low spectral quality of Hyperion data (signal-to-noise ratio (SNR) of $\sim$ 150 in the visible and $\sim$ 60 in the infrared), they have been successfully employed to estimate canopy LAI and leaf chlorophyll content^26^, equivalent water thickness (EWT)^27^, and nitrogen concentration^28^ at the landscape scale. The large archive of Hyperion images has not been extensively exploited, partly due to the lack of field data acquired concomitantly with the imaging, but also due to the extensive preprocessing involved (destriping, desmiling...)^29^. Identifying ways to overcome the lack of corresponding field data or to transfer empirical models from one sensor to another would increase the quantity of training data for satellite missions. As spaceborne imaging spectroscopy missions are becoming more common, it is becoming more possible to build remote-sensing-derived trait databases with their associated environmental conditions, that would subsequently allow continental scale estimates of canopy traits previously unavailable.

**Figure 1:**
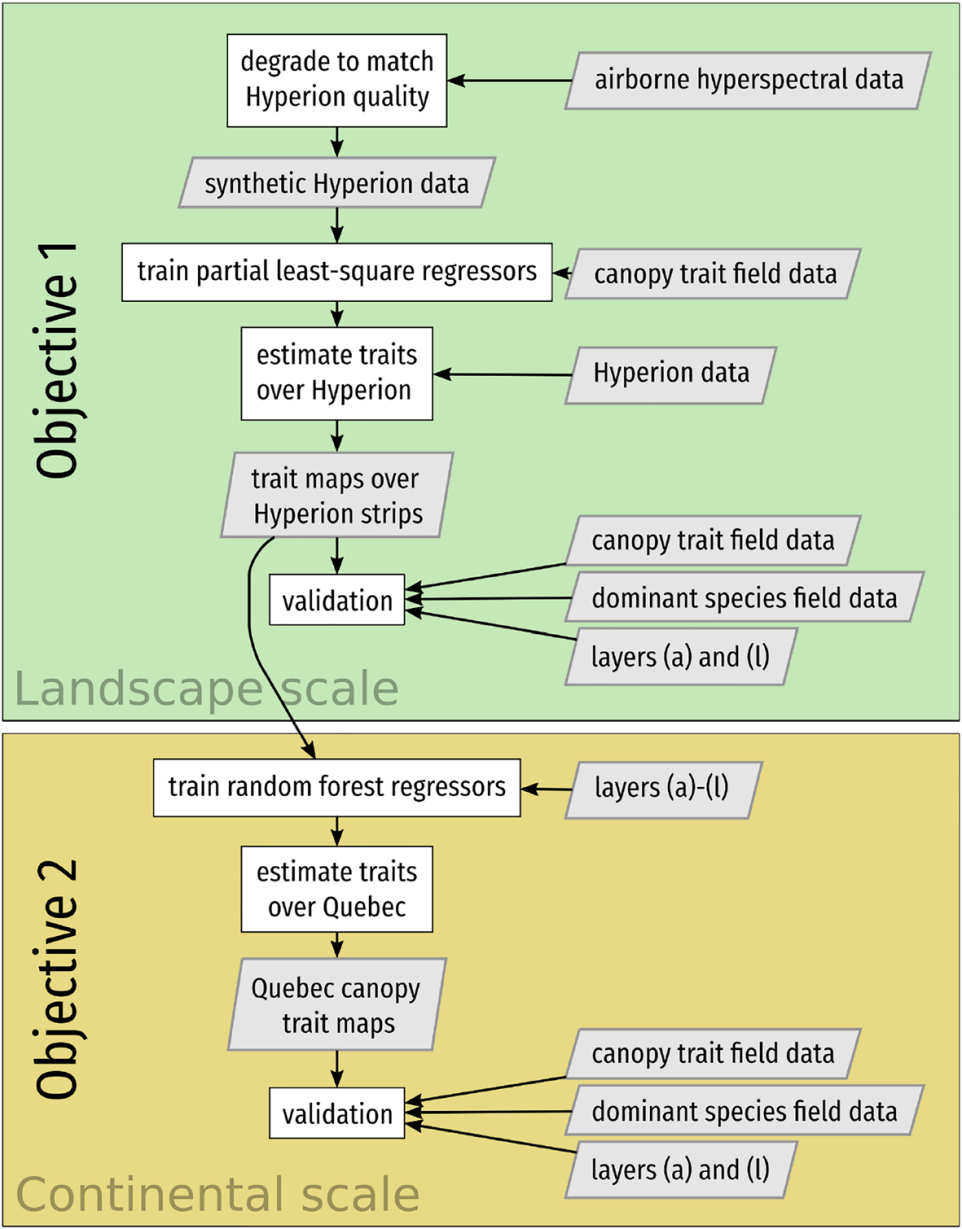
Methodology followed in the present study to obtain Québec-wide canopy trait maps. Layers (a) to (l) are presented in Supplementary Table 1.

Here, we estimate three critical canopy traits from multiple images acquired from the Hyperion sensor. We develop a methodology to exploit data acquired in the context of studies involving other sensors, and utilise them and Hyperion spectra to derive canopy trait estimates and provide a description of vegetation physiology at the continental level over Québec, Canada, with the potential to scale up the method to the global scale (an overview is presented in Fig. 1). We focus on traits with direct links to ecosystem functioning, including the leaf mass per area (LMA), the leaf nitrogen concentration (N) and the leaf carbon concentration (C). LMA affects the relation between photosynthesis and N and is positively correlated with leaf lifespan, higher LMA being required to attain a given lifespan in unfavorable habitats^30^. Leaf nitrogen concentration is closely related to plant productivity and leaf longevity^31,32^, and limitation of photosynthesis by nitrogen increases with latitude^33^. Carbon is mostly present in cell wall components such as lignin or cellulose, and the concentration of C with regards to other elements may be used to differentiate species with different growth and carbon allocation strategies^34^.

**Figure 2:**
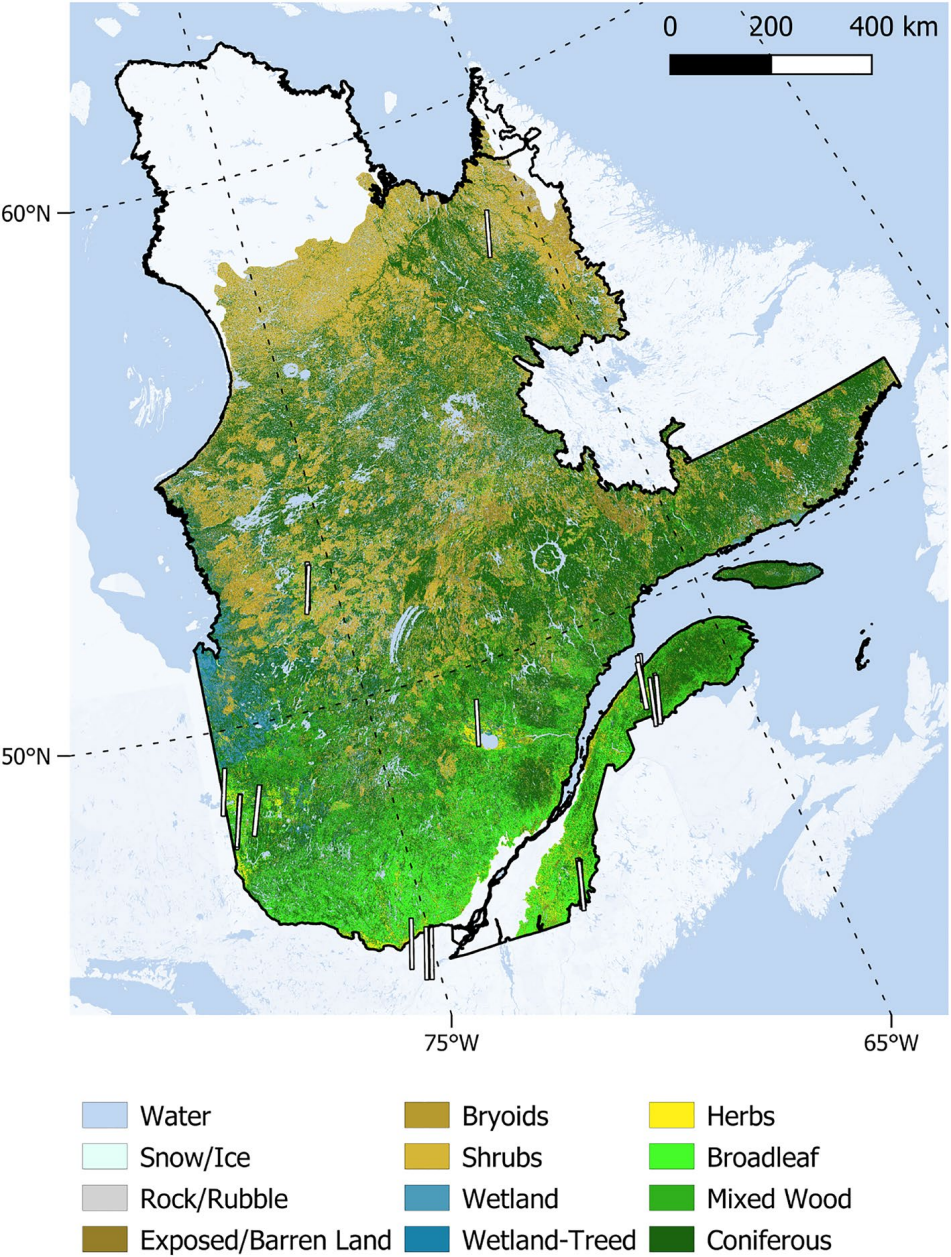
Landcover types of Quebec^36^ and the 25 Hyperion strips used in the present study overlaid in white. Each strip covers about 7.7 $\times$ 42 km. As the latitude increases, forests give way to taiga and tundra.

Our first objective was to assess the capability of models trained using spectral data from different sensors when applied to Hyperion imagery. To do so, we use airborne hyperspectral images and field data acquired over north-eastern USA and Québec to train canopy traits estimators and Hyperion images obtained over Québec during summer (Fig. 2). We test whether it is possible to exploit past and future databases linking airborne remote sensing data with canopy traits in the context of spaceborne imaging spectroscopy by spatially and spectrally degrading the spectra so as to match a satellite sensor’s characteristics. These databases could be used to successfully train models suited for the latter.

Our second objective was to map trait estimates at the continental level. To achieve this, we combine forestry, climatological, and topographical products (see Supplementary Table 1) with the previous estimates from the Hyperion images to extrapolate estimates where imaging spectroscopy data are not available. The biogeographical approach would therefore take full advantage of remote sensing, as not only the layers used for the extrapolation, but also the canopy traits database itself, were obtained through remote sensing. We expect the extrapolation to lead to realistic estimates even over canopies whose dominant species are not represented in Hyperion images. Furthermore, we qualitatively examine the relationships between estimated LMA, N and C to evaluate if they fit ecological expectations about the leaf economics spectrum^35^.

## Results

**Figure 3:**
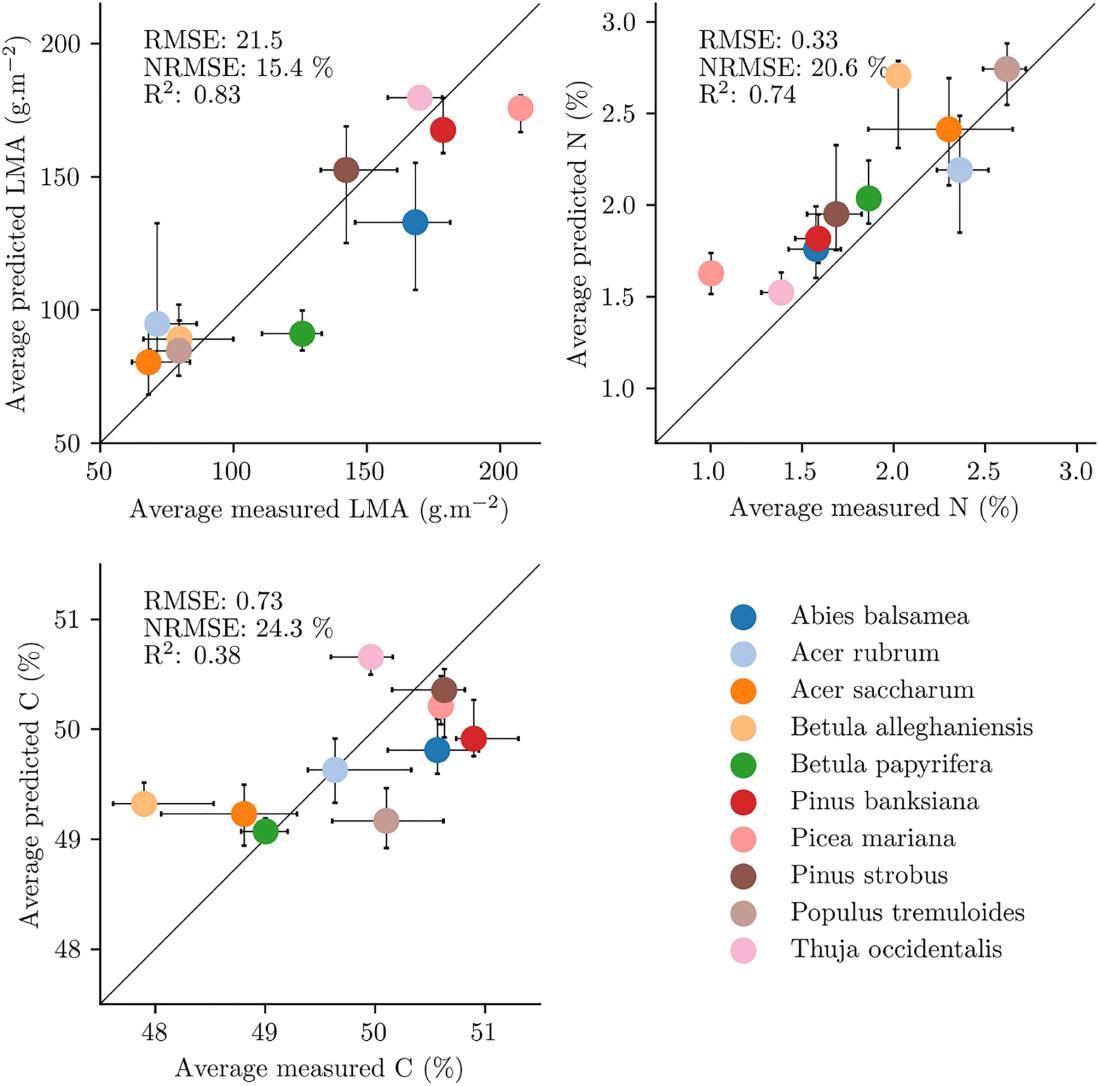
Comparison between the median trait value predicted by the models and the average value measured in the field for tree communities classified by their dominant species in the Hyperion images. The bars, when present, represent the 25th and 75th percentiles for the measured and predicted values.

The training of the estimators on the spatially and spectrally degraded hyperspectral data showed that LMA, N and C could all be estimated with good accuracy (Supplementary Table 2, Supplementary Fig. 3). The estimators were therefore used over the Hyperion data to estimate the canopy traits. The average trait values estimated for each of the dominant species present in the Hyperion strips were in agreement with reference canopy trait values measured in the field during campaigns in north-eastern USA and southern Québec, confirming the transferability of the models from synthetic to real Hyperion spectra. Performances varied depending on the canopy trait of interest: as visible in Fig. 3, NRMSE values of 15.4%, 20.6% and 24.3% were obtained for LMA, N and C, respectively. While the R$^{2}$ scores for LMA and N were above 0.7, that of C was lower with 0.38, as the leaf carbon concentration of *Betula alleghaniensis* (yellow birch) was overestimated. As the estimates derived from the Hyperion images seemed sound, the analysis proceeded further with the upscaling from the Hyperion strips to Québec canopies.

**Figure 4:**
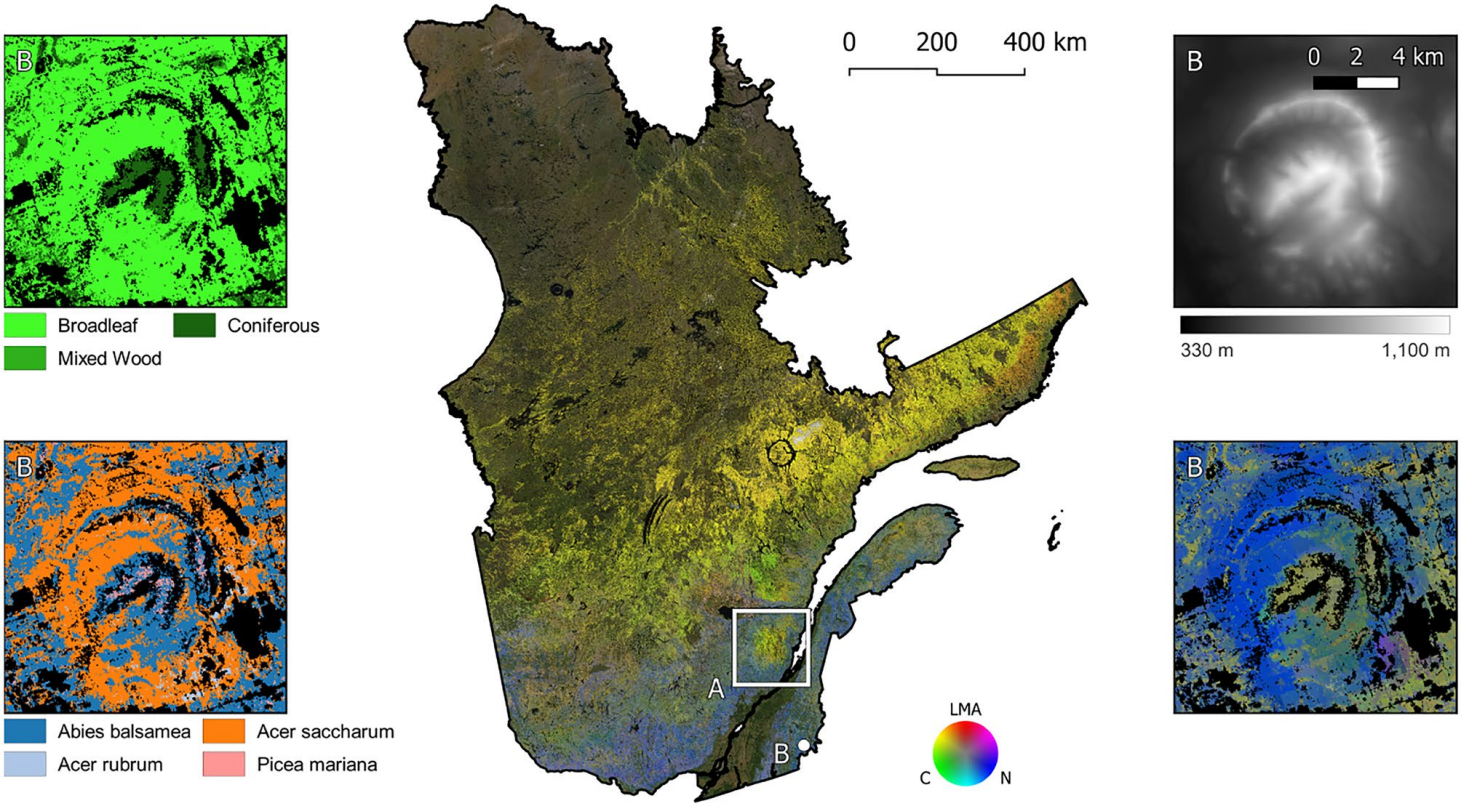
Red–Green–Blue (RGB) colour composites of leaf mass per area (LMA, red), leaf carbon concentration (C, green) and leaf nitrogen concentration (N, blue) of Québec forests with a canopy cover above 30%. Zone A corresponds to the location of the Laurentide Wildlife Reserve, while zone B indicates Mont Mégantic. In the captions, from top left, clockwise: forest stand type^36^, elevation^40^, RGB composite of canopy traits, and dominant species^41^ over Mont Mégantic. In black, areas where canopy traits were not estimated due to low canopy cover or unavailable data.

Figure 4 shows the distribution of the three canopy traits extrapolated across Québec for forest canopies with a $\ge$ 30% canopy closure (CC). The transition from a warm-summer humid continental to a subarctic climate with increasing latitude is evident in our results, with higher LMA and lower N values over northern Québec as the forests change from dominance of broadleaf deciduous to needle-leaf coniferous plant species. Similarly, the Laurentides Wildlife Reserve, a mountainous area characterized by harsh winters and dominated by *Abies balsamea* (balsam fir) and *Picea mariana* (black spruce) in the high plateaus^37^, can be clearly distinguished in southern Québec by its comparatively low N and high LMA relative to neighbouring areas (site A in Fig. 4).

Moreover, the trait predictions capture the effect of climate at smaller scales, as exemplified by the altitudinal gradient at Parc national du Mont Mégantic (site B in Fig. 4). Here, changes in dominant species with increasing elevation are comparable to changes with increasing latitude, where high LMA and low N at high elevation align with the forest transitioning from predominantly *Acer saccharum* (sugar maple) to *Abies balsamea* along the altitudinal gradient^38^—contrary to the identification of black spruce as the dominant in some high elevation areas in layer (a) (Fig. 4, Supplementary Table 1), these areas are in fact dominated by balsam fir, and to a lesser degree *Picea rubens* (red spruce). Additionally, the trait predictions appear to also capture fine-scale, microclimatic trends, with ‘lowland’ coniferous communities with high LMA and low N values at low elevations along streamsides likely characterized by areas of increased soil moisture and cold air drainage^39^. However, the fine-scale variations in trait predictions may also reflect artefacts from the environmental layers (a list is available in Supplementary Table 1), rather than biologically meaningful variation. For example, it is possible to see a low elevation pinkish patch, characterized by high LMA and high N, in the bottom right quadrant of the Mont Mégantic inset in Fig. 4 , even though this area is supposed to be composed dominantly of sugar maples, like its surroundings. It might be that the coarse climatic data corresponding to these pixels did not reflect the local environment.

**Figure 5:**
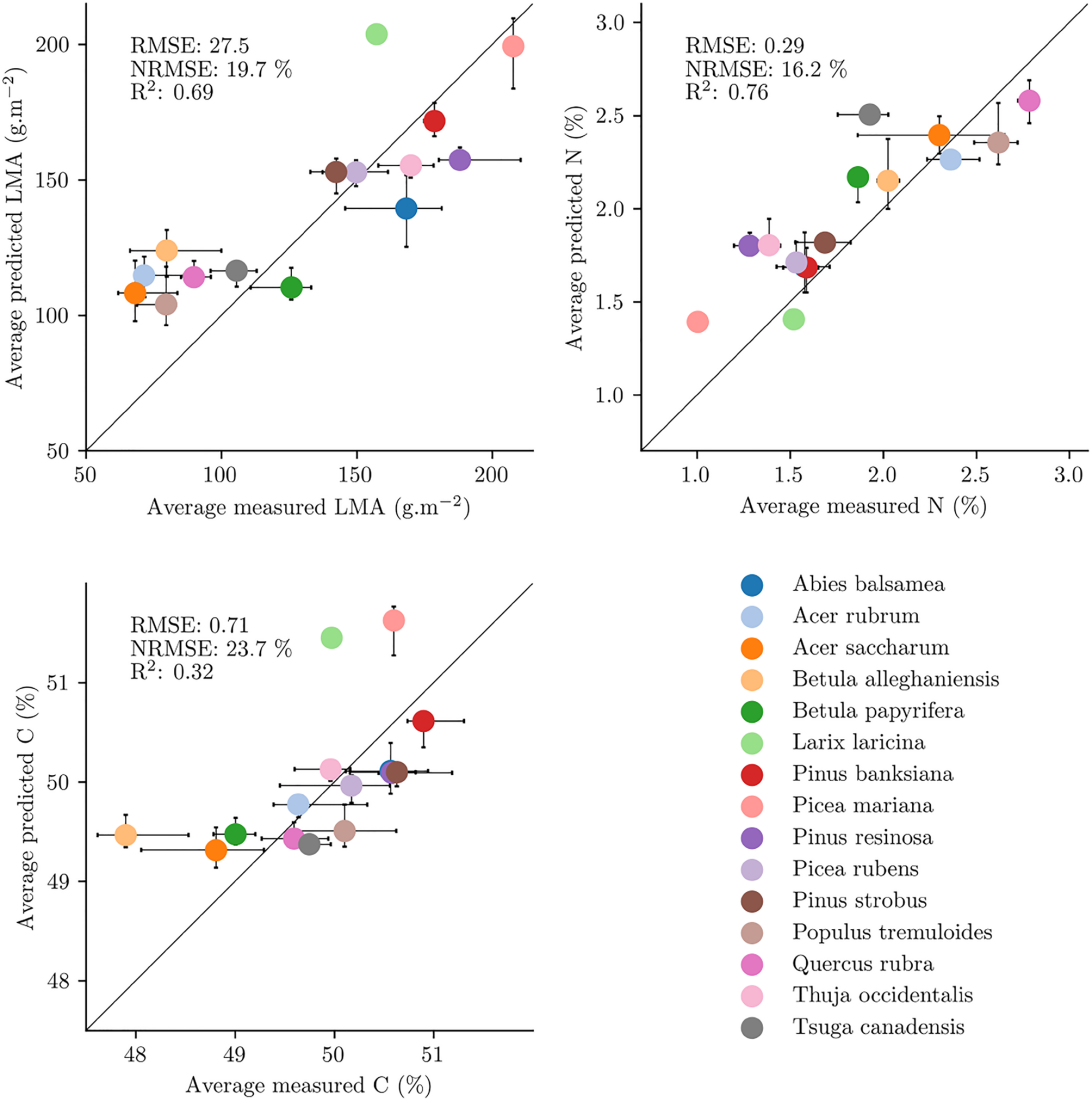
Comparison between the median trait value predicted by the models and the average value measured in the field for tree communities classified by their dominant species in Québec. The bars, when present, represent the 25th and 75th percentiles for the measured and predicted values.

A greater number of dominant tree species occur across Québec than within the Hyperion strips (Figs. 3 and  5), and therefore extrapolators had not been trained for some communities. Despite this, at the regional scale, the average trait values estimated for each dominant species were also in good agreement with those measured in the field, with NRMSE values of 19.7%, 16.2%, and 23.7% for LMA, N and C, respectively. Overall, there was a tendency to overestimate low LMA values compared to estimates directly obtained from the Hyperion images, with estimates for broad-leaved dominated communities going from the 80–100 g m$^{-2}$ range to 100–120 g m$^{-2}$. We found C was overestimated in: *Betula alleghaniensis*, *Larix laricina* (eastern larch), and *Picea mariana*. While *Betula alleghaniensis* was already overestimated over the Hyperion strips and *Larix laricina* was not represented, *Picea mariana* was previously well estimated.

**Figure 6:**
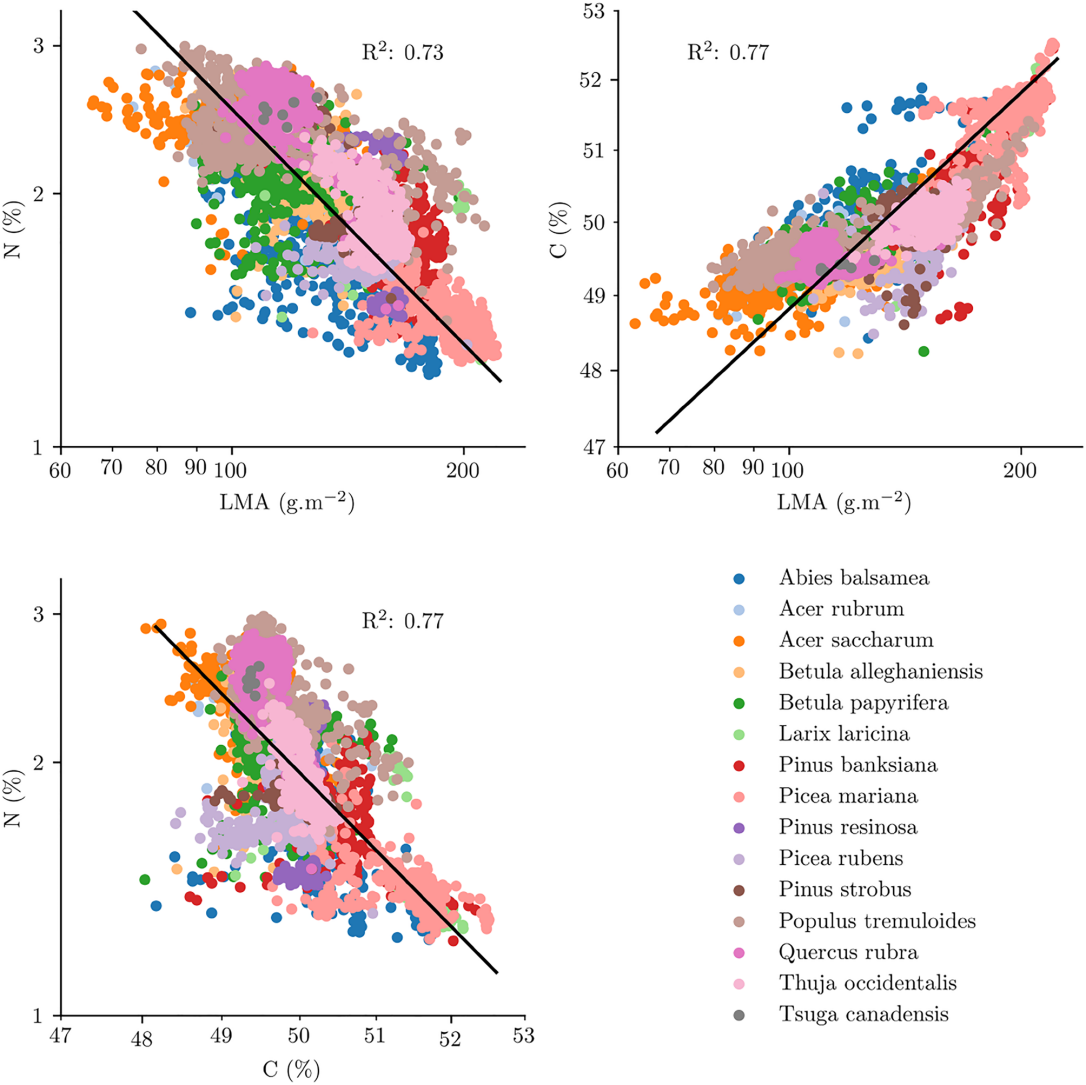
Correlations between the leaf mass per area, leaf nitrogen concentration, and leaf carbon concentration values estimated over Québec.

Figure 6 shows the correlation between the traits estimated over Québec on a pixel-per-pixel basis at a 30 m resolution. After log-scaling of the variables, relationships in line with the leaf economic spectrum^35^, which proposes that leaf investment strategies of nutrients and minerals are distributed over a specific pattern of trait correlations, were visible. Indeed, for each pixel, N and LMA were inversely correlated, with an overall R$^{2}$ of 0.73, and species were heterogeneously distributed over the whole range. Carbon concentration and LMA were also strongly positively correlated, with an R$^{2}$ of 0.77, which translated into a strong relationship between N and C.

## Discussion

Retrieval of leaf functional trait estimates from imaging spectroscopy are most often done at the landscape scale using airborne platforms based on more limited site- and sensor-specific datasets^42^. Moreover, methods for remote sensing of LMA and N are still under development^11^, and scaling LMA from the landscape to the regional scale remains a challenge^20^. One reason for this gap is the lack of availability of regular, large-scale imaging spectroscopy data. Another reason is the difficulty in obtaining representative reference field data to train and validate the estimators, as the collection of such data is very time-consuming and less standardized, with few global validation datasets^43^. Moreover, the absence of a standard field data collection protocol may lead to variations in the reference field values: for instance, the use of community-weighted means or the vertical sampling of the canopy will affect how much the inter- and intra-specific variability are taken into account, as well as the overall measurement uncertainties^43,44^. Our method allows the estimation of three key traits using hyperspectral imagery from AVIRIS, CASI and SASI, and Hyperion, with retrievals in line with observed ranges of field-measured values across forest species. This demonstrates that images from various imaging spectrometers can be used to train estimators dedicated to acquisitions from another, different spectrometer. Moreover, the ability to exploit both field and imaging data from previous remote sensing campaigns would help to capture traits variability of mapped ecosystems for reference and accuracy assessment purposes. The present study used relatively straightforward spatial and spectral resampling methods to exploit this auxiliary data, which may have limited the final accuracy of the estimators. More refined spatial response resampling methods are being developed to simulate data for and from various imaging spectrometers, which should lead to more realistic synthetic databases^45^. In the context of the new satellite hyperspectral missions, this means that previous datasets may be exploited to improve the generalizability and accuracy of the estimators over the variety of environments mapped by the new sensors.

As imaging spectroscopy data are not yet available at the continental scale, an extrapolation method is necessary in order to map the canopy traits. For a given community, community weighted trait values depend on the age of the stands^46,47^, their health^48^, and local climate^49^. Several regional and global products derived from multispectral data and global climate models are available and can be used to extrapolate local estimates of these variables^22^. However, an inherent limitation is that the data with which to undertake the extrapolation may have errors (e.g. species occurrence in incorrect places), and that the data available for training may not fully reflect the range of conditions encountered in the mapped area (in the present study, a greater variety of species dominated canopies across Québec than across the areas covered by the Hyperion strips). Overall, these limitations highlight how much a better undertanding of error-propagation within extrapolation and scaling methods would benefit large-scale remote sensing vegetation studies. Efforts on this matter are currently undergoing^50^ . Still, it might be that incorrect auxiliary layers in specific areas are of little consequence, depending on the objective of the mapping, as the geographical trends of the canopy traits are captured nevertheless.

The sparsity of the training databases, on the other hand, requires more attention: some estimators might not be able to handle missing features, which could lead to erroneous estimates for cases not within the boundaries encountered during training. In the present study, it was shown that the model estimated values in area of unknown species were still reasonable, but in general, attention should be paid to select estimators able to handle extrapolation. Another way to reduce the sparsity of the training database could be to use more spaceborne sensors to derive the estimates. The present study only used 25 Hyperion images, however two additional spaceborne imaging spectrometers, PRISMA and EnMAP, are now also available and their acquisitions will also be able to be exploited over time. As more areas are imaged, remote-sensing-derived trait databases should only become more exhaustive, which would in turn increase the overall accuracy of global maps.

The final prediction map of the three foliar traits across Québec’s closed-canopy forests captured the turnover from broadleaved to coniferous forests, both at the continental-scale (i.e. latitudinal patterns) and at the local-scale (i.e. altitudinal patterns in Parc National du Mont Mégantic). There is a tight association, phylogenetically and functionally, between the foliar traits examined in this study, and the coordinated differences in trait values driven by the separate phylogenetic histories of broadleaf deciduous angiosperms and evergreen conifers^51^. Functionally, these two phylogenetic groups occupy different positions along the acquisition-conservation continuum linked to the leaf economics spectrum, with coniferous species characterized by trait values associated with more conservative functional strategies^35,52^. In line with this, we found that land cover type, which distinguished broadleaved and coniferous stands, was the most important predictor of the three traits for un-mixed stands (Supplementary Fig. 4).

While climate variables were the second most important predictors for un-mixed stands, their importance was relatively small compared to landcover type, suggesting that climate has its strongest impact indirectly, via its influence on deciduous vs. coniferous vegetation^51,53^. However, in mixed stands, where both clades are present, climate variables were the most important predictors of canopy traits, indicative of a direct effect. Climate has long been considered the primary driver of species composition, with a continuous transition from broadleaved temperate forest to coniferous boreal forest with increasing latitudes and elevation^54^. Additional non-climatic factors, such as soils, topography, and disturbance, can modulate the effect of climate and result in fine-scale heterogeneity in species composition^54^. The moderate importance of dominant species as a predictor variable in both un-mixed and mixed stands may be due to the effects of these non-climatic drivers. However, fine-scale patterns in predicted traits should be interpreted with caution, as exemplified by Parc national du Mont Mégantic, where some fine-scale variations appear to capture patches of low elevation coniferous stands, while others do not have a clear biological interpretation (based on our extensive knowledge of this area).

Over Québec, LMA, N, and C estimates for each pixel showed patterns coherent with the leaf economic spectrum^35^: N was negatively correlated to LMA, while C, as the primary component of the leaf structure, was positively correlated to LMA (Fig. 6). Obviously, LMA, N, and C are connected, as both N (through proteins) and C (through lignin, cellulose, hemicellulose, sugars and starchs) are both major components of the LMA^55^. However, these subcomponents present different spectral features, and their concentrations vary depending on the species and their ecological strategies^56,57^. Estimators for LMA, N and C were trained independently, and as a result had different variable importance results across the VSWIR range (see Supplementary Fig. 5), although the spectral regions identified as important were more similar between LMA and C than with N. This similarity is likely due to the fact that C is the major component of LMA (i.e. around 50%^4^). Conversely, the spectral features used by the model to estimate N focused more on specific regions of the infrared, with two peaks notable around 1530 and 2300 nm, close to known protein/nitrogen absorption bands^58,59^. Given that these three estimators were trained independently and selected different regions of the spectrum to derive traits *a priori* uncorrelated, further confidence can be put in the regional maps.

Environmental associations of community-weighted canopy traits have been used successfully to predict vegetation function^60^, using trait databases such as TRY^61^ or AusTraits^62^. However, compiling these databases requires a massive collective effort in obtaining a sufficient quantity of trait $\times$ species combinations to compensate for the sparsity of the data matrices^63^. Using canopy traits measured through imaging spectroscopy allows us to capture these combinations over a large diversity of communities and environmental conditions at minimal additional cost, which would further our understanding of plant function and trait phenology^64^. Moreover, imaging spectroscopy would allow for continuous updating of trait estimates over time and space, and to track vegetation response to disturbances.

*Conclusion* We show that models trained on synthetic data and Hyperion images can be used to map leaf structural and biochemical traits over large landscapes. The subsequent estimates can be used conjointly with regional data products to produce maps of canopy traits. We estimated LMA, N and C values of Québec forests at a 30 m spatial resolution over 237,604 km$^{2}$ and assessed the accuracy of the estimates depending on the dominant species of each community using reference field data. We showed that the resultant maps were in accordance with the leaf economic spectrum at the continental scale. Despite the limitations of Hyperion, our method was still successful in mapping the traits over Québec, demonstrating the high potential of the recent and future spaceborne imaging spectrometers to further predictive ecology and biodiversity conservation efforts (see Table 1 for SNR comparisons).

**Table 1:** Signal-to-noise ratio (SNR) of three spaceborne imaging spectrometers: Hyperion, PRISMA, and EnMAP.

Sensor	SNR		Reference
	VNIR	SWIR	
Hyperion (50% reflectance)	$\sim$ 150	$\sim$ 60	^65^
PRISMA (30% reflectance)	160–200	100–800	^66^
EnMAP (30% reflectance)	> 400	> 180	^67^

*VNIR* Visible near infrared, *SWIR* Shortwave infrared.

## Methods

*Hyperspectral data* We used airborne hyperspectral data acquired over multiple campaigns in Québec and north-eastern USA, either by the Compact Airborne Spectrographic Imager 1500 (CASI-1500, referred to as CASI in this study) and Shortwave infrared Airborne Spectrographic Imager (SASI-600, referred to as SASI in this study) sensors or by Airborne Visible/Infrared Imaging Spectrometer (AVIRIS). Each imagery underwent different processing steps depending on the sensors.

The CASI and SASI images were acquired over Mont Mégantic, Québec, on July 18th 2019. CASI acquired images had a 1.5 m resampled pixel size and was set to 288 bands mode a 2.39 nm full width at half maximum (FWHM) over the 375–1061 nm spectral range, while SASI images had a 2.7 m resampled pixel size and a 15 nm FWHM over the 957–2442 nm spectral range. Surface reflectance was retrieved with ATCOR4 (rugged terrain module), using a Lambert + Statistical-Empirical method for CASI and a Modified Minnaert method for SASI to perform the topographic correction^68^. CASI images were spatially degraded to 2.7 m using a gaussian filter with a 2.7 m FWHM and CASI and SASI images were co-registered using manually selected ground control points. Finally, the SASI bands were used in the overlap region and the remaining CASI and SASI bands were stacked into one hypercube.

The AVIRIS images were acquired over north-eastern USA during the summers 2008–2011. The images cover the 350–2500 nm spectral range at a 10 nm FWHM and a pixel size ranging from 12 to 18 m. Images were atmospherically corrected with ATREM^69^ and topographically corrected using a modified sun-canopy-sensor procedure^70^. The acquisition and processing protocol of the AVIRIS images is further described in the associated study^17^.

As the CASI-SASI and AVIRIS hypercubes’ spectral FWHM, resolutions, and SNR, differed from those of Hyperion, further preprocessing was necessary. All hypercubes were convolved with a gaussian filter with a 10 nm FWHM, resampled to Hyperion’s spectral bands, and a wavelength-dependant gaussian noise was added to the spectra so as to match Hyperion’s SNR and obtain Hyperion-like spectra. A comparison between Hyperion and Hyperion-like vegetation spectra is given in Supplementary Fig. 1.

We obtained the Hyperion images at the radiance level from the US Geological Survey data portal (earthexplorer.usgs.gov). Due to poor calibration of the pushbroom sensor, Hyperion images contain artefacts (low SNR, stripes, spectral smile...) that have to be accounted for before further processing. In order to obtain surface reflectance, Hyperion images were preprocessed using SUREHYP^71^. SUREHYP is a Python package bringing together multiple methods for destriping, desmiling, and performing atmospheric correction so as to facilitate the processing of a large number of Hyperion images. Surface reflectance was obtained using the SMARTS model^72,73^, using the Canadian Digital Elevation Model from Google Earth Engine (GEE) to perform a topographic correction.

We only retained images acquired from June to August (corresponding to peak greenness) with less than 20% cloud cover, and rejected images containing too many cirrus clouds based on visual assessment, leaving a total of 25 images for this study (Fig. 2 and Supplementary Table 4).

Finally, we smoothed Hyperion and Hyperion-like spectra with a five-band, second order Savitzky-Golay filter, and spectral bands above 2000 nm received an additional smoothing with a nine-band, second order Savitzky-Golay filter. We selected the spectral ranges 500–750 nm, 790–890 nm, 980–1104 nm, 1200–1310 nm, 1508–1760 nm, and 2100–2300 nm as appropriate for the study as they are outside of common atmospheric absorption features.

*Field data collection* Canopy traits were obtained over 221 plots across north eastern USA and Québec.

Over north eastern USA, canopy traits were obtained over sites in multiple states (Maryland, New York, Wisconsin, Michigan, and Minnesota), sampling 36 dominant species. Foliage was collected from the top, middle, and bottom sections of the canopy of the species of each plot, and traits were measured through reflectance spectroscopy. More details regarding the canopy trait measurements and their upscaling to the canopy level are given in the associated study^17^. The plot-scale foliar chemical and morphological traits are available online^74^.

Over Québec, canopy traits were quantified on mature, fully sunlit, healthy leaves harvested from individuals within two provincial parks in southern Québec, Parc national du Mont Mégantic and Parc national du Mont Saint Bruno, during the 2018 and 2019 growing seasons. Harvested leaves were bulked per individual with a minimum of 10 individuals sampled for common and 5 individuals sampled for rare tree species (see^75,76^). The canopy trait measurements followed the standardized protocols developed by the Canadian Airborne Biodiversity Observatory (CABO). In brief, bulk leaf samples were rehydrated for 6 hrs and scanned to quantify total leaf area (LA, in cm$^{2}$; CanoScan LIDE 220 scanner, Canon, Brampton, Canada and WinFOLIA Reg 2016b software, Regent Instruments Inc., Québec, Canada). Leaves were then oven-dried at 65°C for $\ge$72 hrs and weighed to determine leaf dry mass (LDM, in g). We calculated LMA as leaf dry mass divided by leaf area (LMA = LDM $\times$ LA$^{-1}$ $\times$ 10$^{4}$)(see^77^). Leaf carbon and nitrogen concentrations (mass-based %) were quantified on oven dried and ground bulk leaf samples using an elemental analyzer (CHNOS Elemental Analyser Vario Micro Select, Elementar Analysesysteme GmbH, Hanau, Germany)(see^78^). Canopy traits were averaged per species.

We calculated the community-weighted means (CWMs) of each canopy trait as the sum of the average species’ trait values weighted by species’ relative abundance for 65 forest inventory plots (circular plots with 15 m radii,  706 m$^{2}$) distributed across Parc national du Mont Mégantic (n = 50) and Parc national du Mont Saint Bruno (n=15)(see^79^). Species’ relative abundance per plot was calculated as species’ crown area as-visible-from-above divided by the total crown area as-visible-from-above^80^.

Nitrogen and carbon concentrations (mass-based) were preferred over contents (area-based) as traits of interest, as laboratory measurements are usually closer to mass basis values, and nitrogen concentration is commonly used as an indicator of leaf litter quality^81^. Moreover, converting to area would have meant combining two measurements (trait concentration and leaf mass per area) together with their uncertainties, while using only mass-based traits allows to separate models with their respective uncertainties. A summary of the trait statistics is given in Supplementary Table 3. Permission was obtained when necessary to collect the plant materials, and collection was done in accordance with local and national regulations. For the Québec sites, formal identification of the plant material was done by members of the CABO teams. Plant specimens will be deposited at the Marie-Victorin herbarium of the Université de Montréal.

*PLSR training and application* We used PLSR to estimate LMA, N and C from the hyperspectral images. PLSR has been successfully used on several occasions to estimate canopy traits from hyperspectral imagery over forests, grasslands, crops, or biomes^16–18,82,83^. Although it is quite flexible, PLSR may underperform when the distribution of the target variables used for training present a significant skewness . As this was the case for LMA, out of the 124 database entries with LMA values lower than 100 g m$^{-2}$, we undersampled the lower end of the database. Fifty values were randomly selected and retained when developing models dedicated to LMA. Nitrogen and C did not present such skewness and the whole database was used for model development (Supplementary Fig. 2). All trait data were further transformed so as to present a normal distribution prior to model training.

The database was separated into a training set representing 70% of the entries, and a validation set representing 30%. First, using the training set, PLSR model were trained with an increasing number of latent variables. For each model, a 50-fold cross-validation was done, and the mean regression coefficient for the testing subsets (Q$^{2}$) was saved. The optimal number of components was determined as either (i) the number of components for which the first local maximum of Q$^{2}$ was observed or (ii) in the event of a constantly increasing Q$^{2}$, the number of components after which the relative increase was less than 5%^84^.

The final ensembles of PLSR models were trained using the optimal number of components. The ensembles were made of 500 models, each model being trained with 70% of the training set. Their accuracies were subsequently assessed using the independent validation sets.

We then estimated LMA, N, and C over the forested areas of the 25 Hyperion images. These areas were identified using publicly available Québec-wide landcover and canopy cover maps derived from Landsat imagery^36,85^. The European Environmental Agency defines a forest as a treed area with trees higher than 5 m and a canopy cover above 30%^86^: in the present study, forested areas were associated with the ‘coniferous’, ‘broadleaf’, and ‘mixed wood’ pixels having at least 30% canopy cover. As validation data was not available with the Hyperion images, accuracy assessment of the estimates could not be direct.

To assess the accuracy of the estimates over the Hyperion images, we classified tree communities depending on their dominant species (derived from layer (a), see Supplementary Table 1) and computed the distribution of the estimated canopy traits for each dominant species. Then, using the dominant species data sampled in the field, the same was done with the canopy traits measured over Québec and north-eastern USA. We then compared the statistics for each dominant species, and determined NRMSE, RMSE and R$^{2}$ by using the median value of each distribution in order to assess whether or not the trait variations between dominant species had been captured. When Hyperion images overlapped, the estimated value was set to the average of the estimated values from each image. We only used non mixed pixels for validation purposes: pixels marked as ‘mixed wood’ and for which the confidence in the predicted dominant species was below 0.5 were excluded from the validation (using layer (l) in Supplementary Table 1).

*Extrapolation of the estimates* We used random forest (RF) models in order to upscale our trait estimates. The 1 km resolution climatological data (average minimum and maximum temperatures, average precipitations) from the AdaptWest Project^87^ (available at adaptwest.databasin.org), as well as 30 m resolution satellite forest information (land cover; species; canopy cover; stand height; stand aboveground biomass; stand basal area; stand stem volume, available at opendata.nfis.org)^36,85^^41^ and the elevation from the Canadian Digital Elevation Model resampled to 30 m were used as input samples, and the values estimated from the Hyperion strips at a 30 m resolution as target samples. Supplementary Table 1 gives a summary of the predictor layers used in this study.

Two RF models were trained for each canopy trait, one for non-mixed stands (e.g. fully coniferous or fully broadleaved) and one for mixed stands. The training sets represented 70% of the data available and the validation sets 30%, and the optimal hyperparameters of the RF models were determined through grid searches. The models were subsequently applied Québec-wide to estimate canopy traits and RMSE, NRMSE, and R$^{2}$ scores between measured and predicted values were again used to assess estimates accuracy.

### Supplementary Information

Supplementary Information.

## Data Availability

The datasets used and/or analysed during the current study are available from the corresponding author on reasonable request.
