# Supplementary material for: Mapping canopy traits over Québec using airborne and spaceborne imaging spectroscopy
Source: Sci Rep. 2023 Oct 11;13:17179. doi: 10.1038/s41598-023-44384-0 (PMC10567784; doi:10.1038/s41598-023-44384-0)
Supplement: Supplementary file 1 — Supplementary Information. [file 41598_2023_44384_MOESM1_ESM.pdf]

# Québec-wide layers used

**Table 1**: Québec-wide supplementary layers used for this study. When necessary, layers were resampled to 30 m.

| Layer | Type | Original resolution | Ref. | Source |
|---|---|---|---|---|
| (a) | dominant species | 30 m | [41] | |
| (b) | landcover type | 30 m | [36] | opendata.nfis.org |
| (c) | canopy cover | 30 m | [84] | opendata.nfis.org |
| (d) | average canopy height | 30 m | [84] | opendata.nfis.org |
| (e) | aboveground biomass | 30 m | [84] | opendata.nfis.org |
| (f) | basal area | 30 m | [84] | opendata.nfis.org |
| (g) | stem volume | 30 m | [84] | opendata.nfis.org |
| (h) | average yearly min. temp. | 1 km | [86] | opendata.nfis.org |
| (i) | average yearly max. temp. | 1 km | [86] | opendata.nfis.org |
| (j) | average yearly precip. | 1 km | [86] | adaptwest.databasin.org |
| (k) | digital elevation model | 0.75 arcsec | [40] | developers.google.com |
| (l) | dominant species confidence | 30 m | [41] | |

# Real and synthetic Hyperion spectra

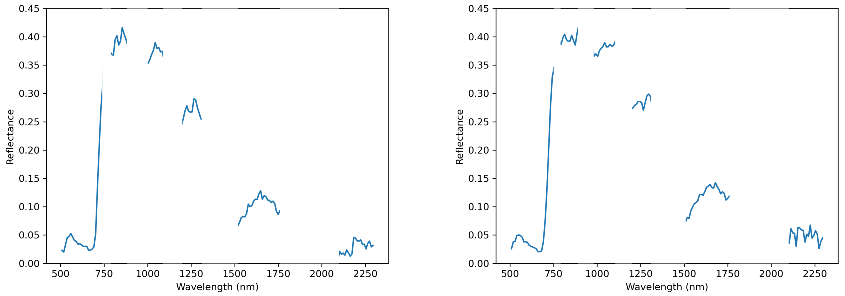


**Fig. 1**: On the left, an example of a Hyperion vegetation spectrum extracted from one of the image. On the right, a synthetic Hyperion vegetation spectrum obtained by degrading spectra acquired by AVIRIS.

# Statistics of the estimators

**Table 2**: Statistics of the partial least-square regressors during training and validation using the synthetic Hyperion spectra. n corresponds to the number of samples used for training and validation, and N corresponds to the number of latent vectors. The train and test subsets of the training set indicate the average RMSE and $R^2$ obtained by the 500 models when trained and tested over 70% and 30% of the training set, respectively. Validation corresponds to the external set not considered during the training phase.

| Leaf Trait | n | N | Training (70% of n) | | | | Validation (30% of n) | |
|---|---|---|---|---|---|---|---|---|
| | | | $RMSE_{train}$ | $R^2{}_{train}$ | $RMSE_{test}$ | $R^2{}_{test}$ | RMSE | $R^2$ |
| LMA ($g.m^{-2}$) | 147 | 2 | 32.0 | 0.55 | 34.0 | 0.52 | 20.3 | 0.79 |
| N (%) | 221 | 6 | 0.28 | 0.73 | 0.32 | 0.68 | 0.25 | 0.82 |
| C (%) | 221 | 2 | 0.95 | 0.30 | 0.98 | 0.28 | 0.94 | 0.53 |

# Trait values comprised in the databases

**Table 3**: Summary statistics of the canopy traits measured over the Mont Mégantic and north-eastern USA plots that were considered to train and validate the partial least-square regressors (221 entries).

| Leaf Trait | Min. | Max. | Mean |
|---|---|---|---|
| LMA ($g.m^{-2}$) | 45.8 | 264.1 | 109.3 |
| N (%) | 0.96 | 3.29 | 2.10 |
| C (%) | 46.6 | 53.4 | 49.5 |

## PLSR training data

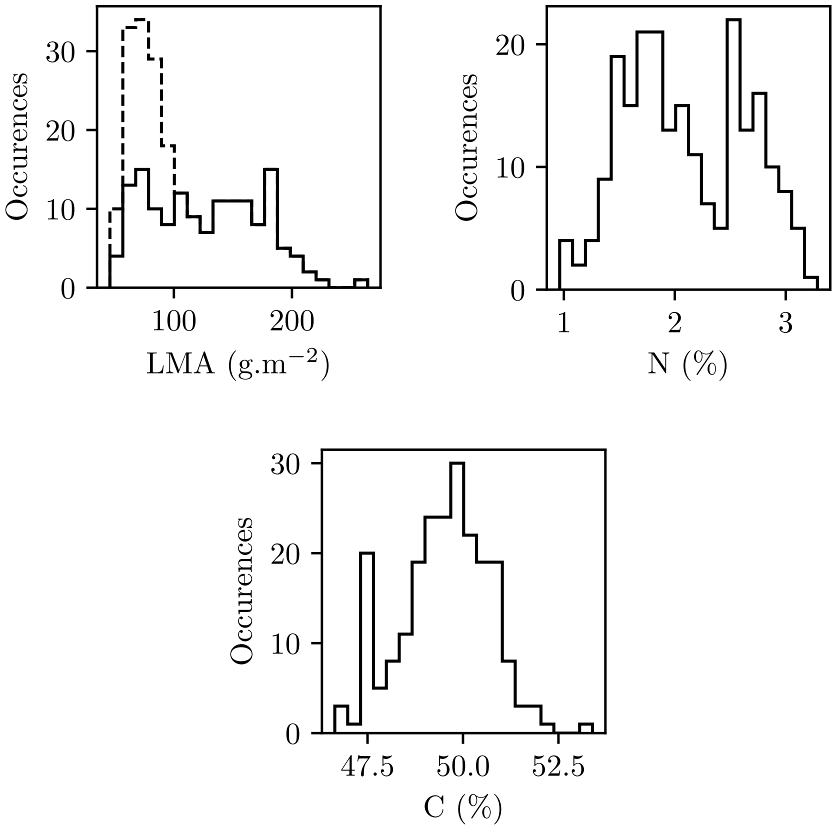


**Fig. 2**: Distribution of the canopy traits used to train the partial least-square regressors models. For leaf mass per area (LMA), the dashed area represents the initial distribution before undersampling to remove the skewness.

# PLSR residuals

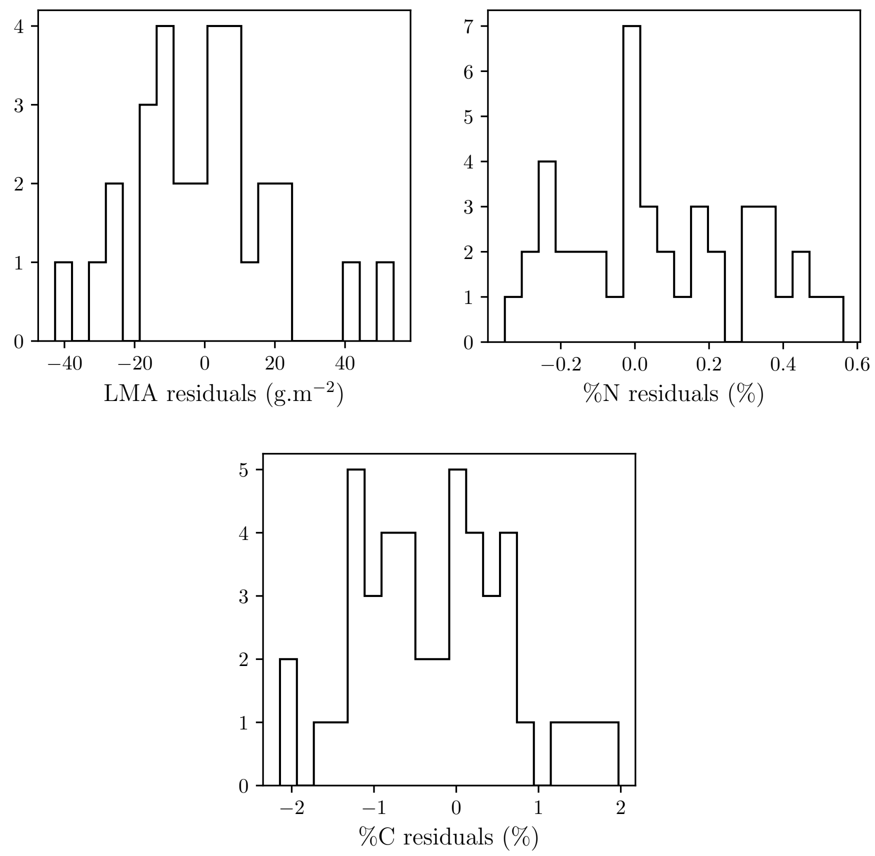


**Fig. 3**: Histograms of the PLSR residuals across fitted values.

# Feature importance for the extrapolation

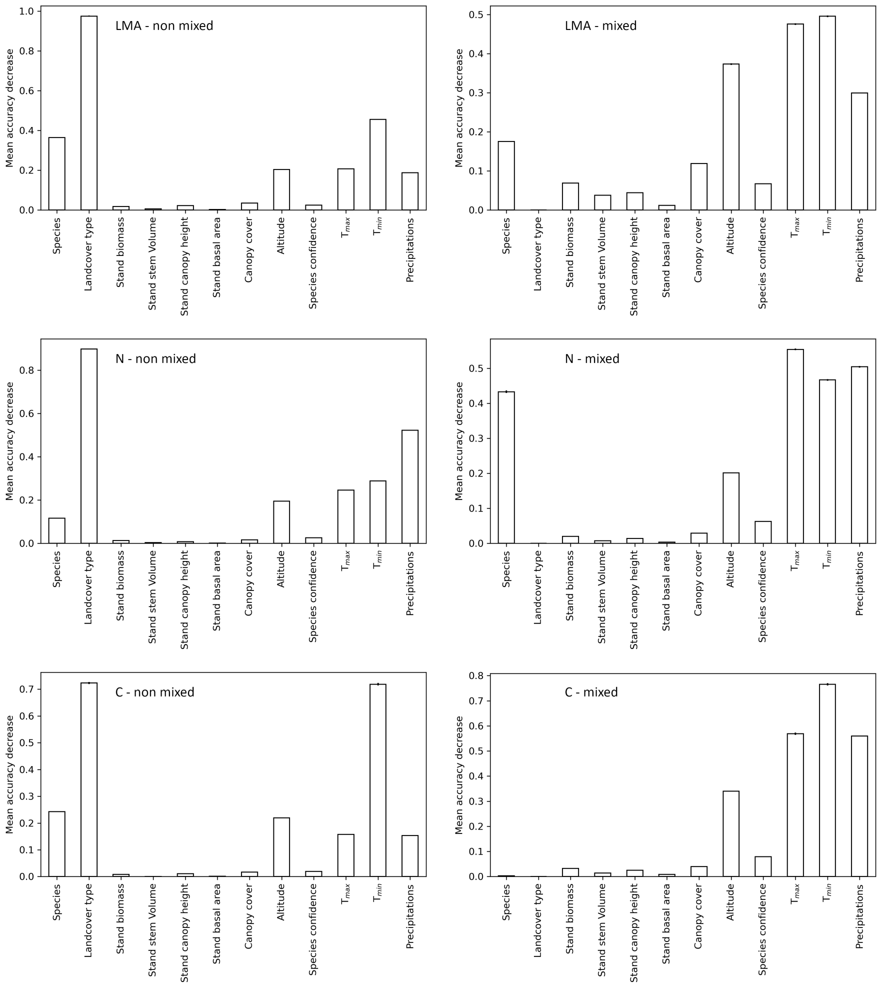


**Fig. 4**: Feature importance after training the random forest regressors to predict the canopy traits estimated from the Hyperion images using auxiliary remote sensing data (see Supplementary Table 1) as inputs.

# Hyperion images

**Table 4**: ID of the Hyperion images used in this study.

| | | |
|---|---|---|
| EO1H0190262015205110K3 | EO1H0190262014172110K3 | EO1H0170242011152110KF |
| EO1H0190262015194110K4 | EO1H0190262011168110K4 | EO1H0150282005193110PV |
| EO1H0190262015186110K3 | EO1H0190262010220110KF | EO1H0150282004175110PZ |
| EO1H0190262015178110K3 | EO1H0180262011228110KF | EO1H0150282004159110PF |
| EO1H0190262014180110K3 | EO1H0170242012209110KF | EO1H0140262002162110PZ |
| EO1H0130282013236110KF | EO1H0130282013212110KF | EO1H0110262016214110KF |
| EO1H0130282013218110KF | EO1H0130202005234110KF | EO1H0110262016178110KF |
| EO1H0110262016176110KF | EO1H0110262016170110KF | EO1H0110262016254110KF |
| EO1H0110262014192110PF | | |

## Variable Importances in the PLSR

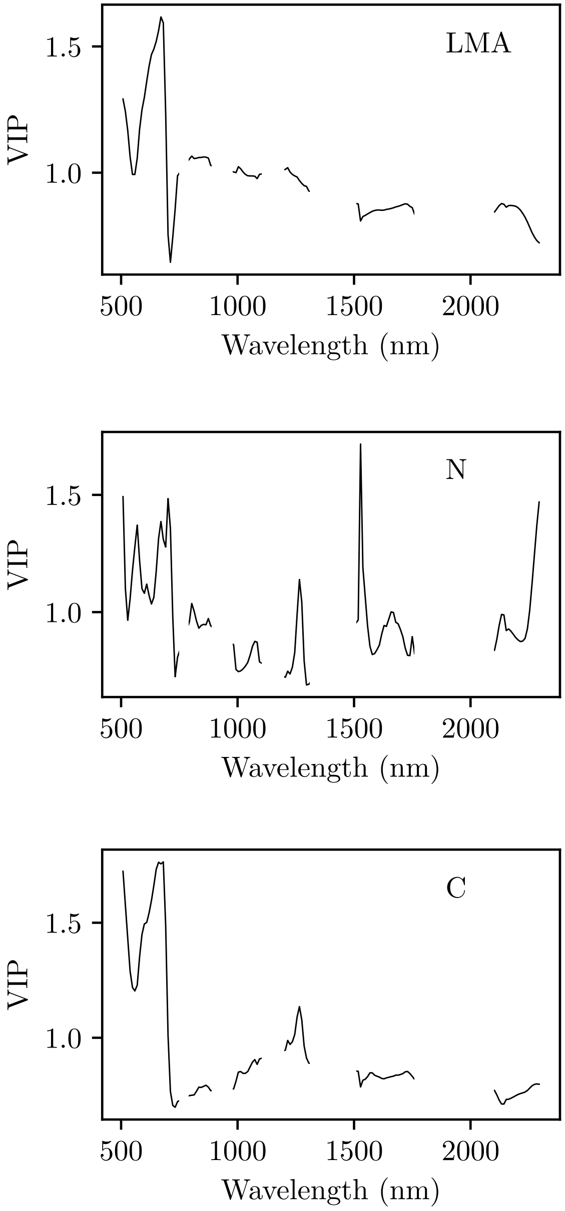


**Fig. 5**: Variable importance in projection in the partial least-square regressors after training.
